# P-2156. Investigating the Relationship of Frailty and UTIs in Kidney Transplant Recipients

**DOI:** 10.1093/ofid/ofaf695.2319

**Published:** 2026-01-11

**Authors:** Rebecca Unterborn, Liza Creel, Kristine M Erlandson, Maheen Abidi

**Affiliations:** University of Colorado, Denver, CO; University of Colorado, Denver, CO; University of Colorado Anschutz Medical Campus, Aurora, CO; University of Colorado Anschutz Medical Campus, Aurora, CO

## Abstract

**Background:**

Urinary tract infections (UTIs) are the most common infectious complication in kidney transplant (KT) recipients and are associated with high morbidity and mortality. Frailty, a vulnerability to stressors, has been associated with increased risk for UTIs in older adults, and UTIs may contribute to worsening frailty. Despite the significant burden of UTIs on KT recipients, the relationship between frailty and UTIs has not been described in this population. Our goal was to investigate the bidirectional relationship between UTIs and frailty.

**Table 1.**
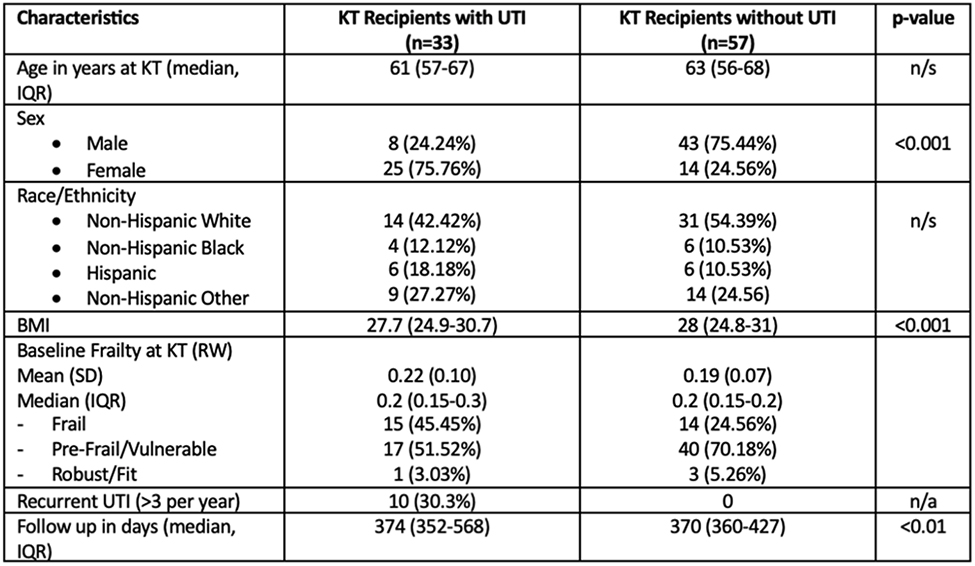
Baseline Characteristics among KT recipients with and without UTIs.

**Table 2.**
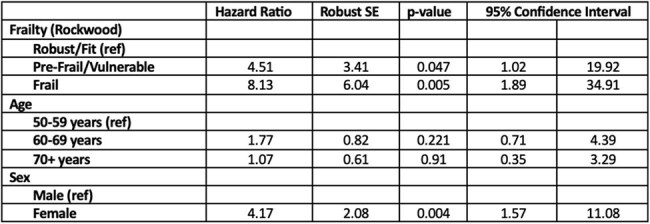
Risk of Developing UTIs according to frailty, adjusting for age and sex.

**Methods:**

We conducted a retrospective chart review of 90 KT recipients, aged ≥50 years, who underwent KT between January-July 2021 and were followed for at least 12 months post-transplant. UTI data was collected at the time of any occurrence; modified Rockwood frailty index (FI) was calculated at each visit with index of ≥0.25 considered frail and ≥0.08 to 0.24 considered pre-frail. We compared the primary outcome of cumulative UTI between the frail and non-frail groups using Cox proportional hazards regression, adjusting for age and sex.

**Figure 1.**
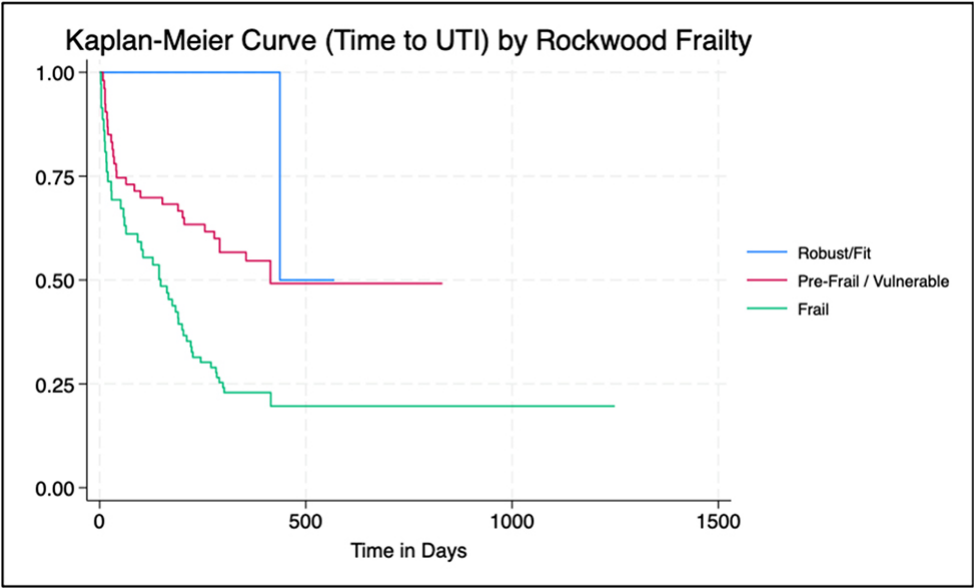
Kaplan Meier cumulative event curves for UTIs according to Rockwood Frailty Index.

**Results:**

33 of 90 (36.67%) KT recipients had ≥1 UTI over the study period. 25 of 33 (75%) identified as female. The median baseline FI was 0.2. Nearly half (45%) of patients with UTIs were considered frail, compared to 25% of patients without UTIs. When compared to non-frail individuals, frail patients were 8 times more likely to have UTIs (HR 8.13, p=0.005) and pre-frail patients were 4 times more likely to have UTIs (HR 4.51, p=0.047). Frail KT recipients also developed UTIs sooner than non-frail patients (Figure 1).

**Conclusion:**

Frailty was associated with earlier occurrence and significantly greater risk of UTIs among KT recipients. Larger studies are needed to better understand the interplay between frailty and UTIs and to facilitate interventions to decrease UTI occurrence among KT recipients.

**Disclosures:**

Kristine M. Erlandson, MD MS, Gilead: Advisor/Consultant|Gilead: Grant/Research Support|ViiV: Advisor/Consultant|ViiV: Travel to meeting

